# Insulator Based Dielectrophoresis: Micro, Nano, and Molecular Scale Biological Applications

**DOI:** 10.3390/s20185095

**Published:** 2020-09-07

**Authors:** Prateek Benhal, David Quashie, Yoontae Kim, Jamel Ali

**Affiliations:** 1 Department of Chemical and Biomedical Engineering, FAMU-FSU College of Engineering, Tallahassee, FL 32310, USA; david1.quashie@famu.edu; 2 National High Magnetic Field Laboratory, Tallahassee, FL 32310, USA; 3 American Dental Association Science & Research Institute, Gaithersburg, MD 20899, USA; kimyo@ada.org

**Keywords:** insulator dielectrophoresis, dielectrophoresis, cell manipulation, biomolecules

## Abstract

Insulator based dielectrophoresis (iDEP) is becoming increasingly important in emerging biomolecular applications, including particle purification, fractionation, and separation. Compared to conventional electrode-based dielectrophoresis (eDEP) techniques, iDEP has been demonstrated to have a higher degree of selectivity of biological samples while also being less biologically intrusive. Over the past two decades, substantial technological advances have been made, enabling iDEP to be applied from micro, to nano and molecular scales. Soft particles, including cell organelles, viruses, proteins, and nucleic acids, have been manipulated using iDEP, enabling the exploration of subnanometer biological interactions. Recent investigations using this technique have demonstrated a wide range of applications, including biomarker screening, protein folding analysis, and molecular sensing. Here, we review current state-of-art research on iDEP systems and highlight potential future work.

## 1. Introduction

Dielectrophoresis (DEP) is the phenomenon in which a neutral but polarizable particle, suspended in a medium, experiences a polarization force generated by an inhomogeneous external electric field. The phrase dielectrophoresis was coined by Herbert Pohl in 1951 to illustrate the translational movement of an electrically polarized particle in a nonuniform electric field [1]. Pohl carried out experiments and published the first journal article on DEP [1]. Pohl′s [1] work clearly highlighted the difference between DEP compared to electrophoresis (EP) and electrothermal flow (EF). In EP, an electrostatic field influences the motion of a charged particle. Depending on the electric field strength and charge distribution at the interface between a particle (with and without an electric double layer) and suspending media (insulating oil or polar solvent), a particle move towards the applied field direction due to induced electrophoretic, electro-osmotic, or slip (“force free”) [2,3]. In EF, electrohydrodynamic-driven temperature gradients induces local conductivity and permittivity changes in fluids to generate forces [4,5], whereas DEP uses nonuniform electrokinetic fields to apply force on uncharged particles. Due to the application of DEP on neutral particles, it became apparent that the same electric fields can be employed to manipulate biological cells. The findings by Pohl paved the way for current research on inhomogeneous electric fields in biology [1,6,7,8,9].

Based on the working principle, DEP is classified as positive DEP (pDEP) or negative DEP (nDEP). If the particle moves in the direction of a higher field gradient, the DEP type is called pDEP, and if the movement is away from high field gradient regions, it is termed nDEP. Both pDEP and nDEP have been utilized to manipulate cells and neutral particles [10,11,12,13,14], with various forms and shapes of electrodes, including planar [15,16], quadruple [17,18], and traveling wave [19,20] designs. The mechanism of particle manipulation using nDEP and pDEP is well understood, and numerous applications have been explored, including cell/particle manipulation [10,11,12], transport [21], separation [22], and sorting [23].

Based on the mode of operation, DEP is categorized as electrode-based DEP (eDEP) [10,11,12] or insulator-based DEP (iDEP) [24,25]. Today, electrode-based DEP is broadly used in microfluidic devices due to its ability to generate high field gradients with low applied voltages. The high gradient fields enable the manipulation of larger size particles, as the DEP force is directly proportional to the electric field strength and volume of the target particles [26]. However, larger gradients in eDEP induce Joule heating [27] and disintegrate electrodes which can lead to the generation of high amounts of toxic species, damaging to cells and cellular organelles [28,29,30]. More recently, iDEP has increased in popularity due to its efficacy towards manipulating cells and maintaining viability [31,32,33]. In iDEP, the electrodes are shielded by an insulating barrier which maintains electrode structure integrity, thereby reducing electrolysis and generation of toxic species. For example, using an iDEP field strength of 2.8 × 10^4^ V/m, mammalian cells were able to maintain viability for up to one hour (with media pH 8), while using eDEP, cell viability is usually limited to tens of milliseconds [34,35].

Electrodeless/insulator-based DEP is a technology where an insulating layer or a structured insulator array is inserted between the electrode and high osmolarity suspending media. The nonuniform electric fields generated by this technique are generally of a lower magnitude than eDEP due to the presence of an insulating barrier between the medium and electrodes. Since the earliest report of iDEP by Masuda et al. [36], it has been generally applied at the micron scale, but there is a growing body of work being carried out at smaller scales. One such example of such a small scale application is biomarker screening [37] and cell response to the drug [13]. Since iDEP can maintain cell viability over extended periods, it is a good candidate to investigate the manipulation of subcellular biology [33].

Over the past 15 years, significant developments have been made in electrode manufacturing processes which have enabled the fabrication of three-dimensional passivated-electrodes [38], 3D printed metallic electrodes [39], and graphene electrodes [40]. These advancements have improved the manipulation efficacy of biological species at low applied electric fields, nurturing the varied frequency range process of microfluidic devices [38,41]. Advanced photolithography technologies, in particular, have paved the way for effectively enabling iDEP to be used in manipulations of cells, viruses, exosomes, and proteins [42,43], thus providing researchers with a handy tool to investigate micro and nanomolecular interactions, and have potential for future use in point-of-care devices. To lay the foundation for a better understanding of potential iDEP applications, we briefly discuss the physical principles of DEP and iDEP; provide the reader with a summary of the state-of-art iDEP work; and recent applications demonstrating manipulation of micro, nano, and molecular level biological materials.

## 2. Theory

**Figure 1:**
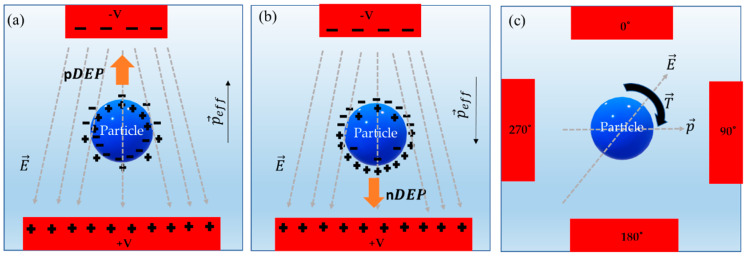
Dielectrophoresis phenomena on a neutral polarizable particle. (**a**) pDEP (particle more polarizable) [44]. (**b**) nDEP (particle less polarizable) [44]. (**c**) Electrorotation [44,57,58].

When an uncharged particle is placed in an inhomogeneous AC electric field, a dipole moment around the particle is generated, causing the particle to polarize and orient in the direction of electric field vectors. At the interface of the polarized particle and the surrounding medium, negative and positive charges accumulate (Figure 1). Due to the application of electric field pulling the charges in opposite directions, an effective dipole moment ($\vec{p}$) is induced on the particle [1,32,33,44]. Hence, from this induced dipole moment, the electrokinetic net forces ($\vec{F}$) and torque ($\vec{T}$) on this polarized particle can be determined as [44]:

(1) $$\vec{F}=\vec{p}\cdot \nabla \vec{E}$$

(2) $$\vec{T}=\vec{p}\times \vec{E}$$

The force relation shows that a particle under an inhomogeneous field is proportional to the magnitude of the applied electric field strength ($\vec{E}$), its divergence, and polarization (P). The effective dipole moment ${\vec{p}}_{eff}$ potential as the increment of electric field distribution can then be defined [44]:

(3) $${\vec{p}}_{eff}=4\pi {\tilde{\varepsilon }}_{m}\left(\frac{{\tilde{\epsilon }}_{p}{}^{*}-{\tilde{\epsilon }}_{m}{}^{*}}{{\tilde{\epsilon }}_{p}{}^{*}+2{\tilde{\epsilon }}_{m}{}^{*}}\right)R^{3}\vec{E}$$

where ${\tilde{\epsilon }}_{m}{}^{*}$, ${\tilde{\epsilon }}_{p}{}^{*}$, and R are the medium permittivity, particle permittivity, and particle radius, respectively. The subscripts *m* and *p* represent the medium and particle, respectively. In an AC field, the complex permittivity is a function of the applied angular frequency (ω) and conductivity of the particle and its surrounding medium. The complex values in Equation (3) are termed as the Clausius–Mossotti (CM) factor ${\tilde{f}}_{CM}$, defined [44]:

(4) $${\vec{f}}_{CM}=\frac{{\tilde{\epsilon }}_{p}{}^{*}-{\tilde{\epsilon }}_{m}{}^{*}}{{\tilde{\epsilon }}_{p}{}^{*}+2{\tilde{\epsilon }}_{m}{}^{*}}$$

Equation (4) is a generally employed CM factor for macro/micron scale particles. In a dielectric, at nano and molecular scales, molecules are marginally conducting and separated by nonconductive spaces from the other molecules [45]. For spherically conducting objects at the molecular scale, Clausius derived the following relationship [45,46]:

(5) $$\epsilon_{m}=\frac{1+2g}{1-g}$$

where $\epsilon_{m}$ is the specific dielectric medium inductive capacity, and *g* is a factor relating the electric field and molecule induced dipole moment within the dielectric [45]. Simplifying Equation (5) and including Lorentz [47] and Lorenz [48] formulation yields the molecular–CM relation [45]:

(6) $$\left(\frac{\epsilon_{m}-1}{\epsilon_{m}+2}\right)\frac{1}{\rho }=\frac{N_{A}\alpha }{3M\epsilon_{0}}$$

where $N_{A}$ is Avogadro’s constant (6.022 × 10^23^), α is the polarizability, M is the molecular weight of the molecules or atoms, ρ is the medium density, and $\epsilon_{0}$ is the free space permittivity [45,48].

The Equations (4)–(6) show the crucial role of Clausius–Mossotti (CM) in understanding the dynamics of biomolecule manipulation. Based on the complex permittivity and conductivity factors of the given analyte, the complexity of the CM equation changes. For example, compared to solid nanoparticles, the hollow shell particle mathematical equation differs [44,49,50]. The complexity of the CM factor increases considerably from micro scale (cells and bacteria) to nano and molecular scale of DNA, proteins, and virions. Some of the known CM factors used in the past are highlighted in Table 1, which shows how the CM factor equations change with respect to the sample. The aspect of complexities involved in understanding the nanomolecular CM factors such as proteins and DNA was succinctly highlighted by Pethig et al. [45,51]. In his article, Pethig [45,51] recommended to reconsider the CM factor for proteins and macromolecules that possess a permanent dipole moment, and recommended by DEP theory, perhaps applicable for highly charged and nonpolar macromolecules [45,51]. Overall, Pethig’s work highlights the importance of evaluating the current understanding of the CM function on nanomolecular scale interactions.

In a nonuniform electric field, the gradient of the net electrokinetic force applied on a polarizable particle causes it to translate towards either higher or lower field regions [44]. For DEP to occur, two conditions are necessary [44]: (1) a variation in the polarizability of the particle and the surrounding medium [44], and (2) an inhomogeneous electric field [44]. In pDEP (Figure 1a), the particle moves towards a region of higher electric field gradient, and if the particle moves away from the higher electric field gradient, then it is termed as nDEP [44] (Figure 1b). The time-averaged DEP force on the dipole is defined as [44]:

(7) $${\vec{F}}_{DEP}=\frac{1}{2}\;V\;{\tilde{\epsilon }}_{m}ℜ\left(\vec{p}\cdot \nabla \;{\vec{E}}^{*}\right)$$

where *V* is the volume of the particle, ℜ is the real part, $\vec{p}$ is the induced dipole moment phasor, and ${\vec{E}}^{*}$ is the complex conjugate of the electric field strength. In inhomogeneous fields, with spatially independent phase, the DEP force simplifies to the form [44]:

(8) $${\vec{F}}_{DEP}=\frac{1}{4}\;V\;{\tilde{\epsilon }}_{m}ℜ\left[\tilde{\alpha }\right]\nabla {\left|\vec{E}\right|}^{2}$$

where $\tilde{\alpha }$ is the effective polarizability of the particle. If we consider a spherical particle, Equation (8) further simplifies to [44]:

(9) $${\vec{F}}_{DEP}=\pi {\tilde{\epsilon }}_{m}R^{3}ℜ\left[{\vec{f}}_{CM}\right]\nabla {\left|\vec{E}\right|}^{2}$$

If the electric field phase is varying spatially, then it produces a nonzero part of the AC electric field. Meanwhile, the dipole remains in a field, and the imaginary part of CM becomes nonzero. The effective dipole moment and electric field interaction generates a rotation or torque on the particle. The phase delay between the dipole moment and the applied electric field results in a change in the path of the electric field vector, causing the dipole moment to align and follow the direction of this applied field vector [44,57,58]. When the applied field is rotating, then it induces rotation on the particle giving rise to DEP torque (Figure 1c). A DEP torque on a spherical particle is defined as [44,57,58]:

(10) $${\vec{T}}_{DEP}=-4\pi {\tilde{\epsilon }}_{m}R^{3}ℑ\left[{\vec{f}}_{CM}\right]{\left|\vec{E}\right|}^{2}$$

where ℑ is the imaginary part. The applied torque is directly proportional to the square of magnitude of the electric field and the imaginary part of the CM factor. The torque on the particle is heavily dependent on the frequency and phase delay of the applied electric field. Measurements below ≈1 kHz often result in fluid electrolysis, leading to the degradation of electrode integrity [54].

## 3. Discussion

Electrode-based DEP has been routinely used to manipulate particles [14,32,59,60] and cells [9,57,61]. Electrodes in eDEP are generally made of electrically conductive materials, such as silver, brass, and gold. A range of methods, from conventional photolithography [62,63] to the latest 3D metal printing [64,65] technologies, for the fabrication of electrodes. The major drawbacks of using eDEP are Joule heating [27,31,32,33], fouling effects [33], and electrochemical reactions that generate toxic species mainly due to electrode disintegration [33,66]. Electrolysis and electro-osmotic effects can further hinder the cell and particle integrity. The drawbacks of eDEP, such as electrolysis and electrochemical effects, can be reduced by using low conductivity media (e.g., <30 mS/m) [33]. This is because in lower conductivity media, the electrode polarization effects can be extended beyond ~5 kHz, which aids in reducing the generation of toxic species and electrolysis effects [33]. However, low electrically conductive media require stronger electric fields to be generated resulting in the utilization of higher current density which increases Joule heating. To overcome the eDEP issues, electrodeless or iDEP has become increasingly popular as electrode disintegration is avoided by shielding the electrodes with suitable insulation. However, it is to be noted that both eDEP and iDEP can suffer from joule heating effects [67].

**Figure 2:**
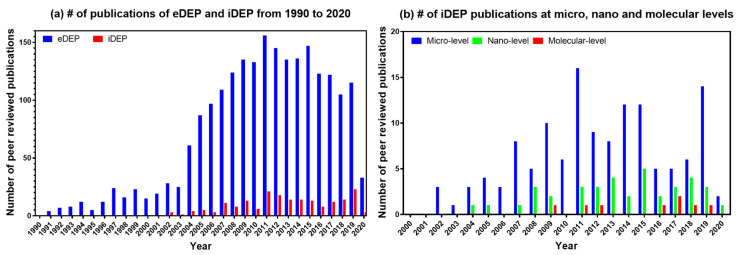
Reports of biological applications of insulator based dielectrophoresis (iDEP) and electrode based dielectrophoresis (eDEP); (**a**) Shows the number of publications reported employing both eDEP and iDEP methods. (**b**) Shows an overall observation of gradual research publication increase for nanomolecular iDEP over the recent year from 2002 to 2020.

To quantify the increasing popularity of the iDEP, we carried out a thorough Web of Science literature survey of eDEP and iDEP from 1990 to 2020 specifically for biological applications. The two methods of DEP in question were designated as “electrode-based” and “insulator-based” or “electrodeless”. The database query returns were reviewed to ensure the subject matter properly matched the query. During the search, the irrelevant returns were disregarded, such as the “N/A” category reserved for nonexperimental submissions and other review articles of DEP. We collected data of peer-reviewed publications excluding conference abstracts and patents. The yearly total since 1990 to 2019 revealed the trend shown in Figure 2. The number of eDEP publications increased exponentially over the period from1990 to 2011. Then, a steady decrease in the eDEP publication trend was seen from 2011 to 2020. The trend in publications related to iDEP showed about a ≈ 16% increase and a ≈ 33% decrease with eDEP during this period. Overall, the plots suggest that the future interest in iDEP is slowly but steadily increasing and shifting towards smaller scale applications.

**Figure 3:**
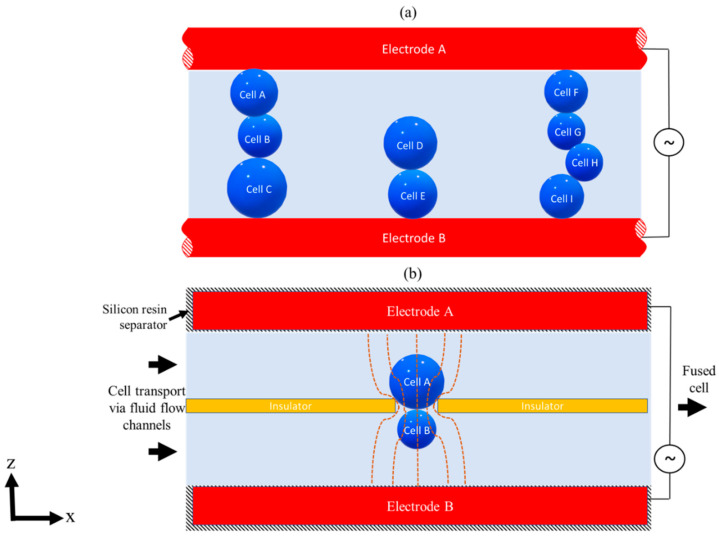
eDEP and iDEP concept sketch. (**a**) Figure showing side view concept sketch of eDEP showing pearl chain formation of cell in a higher magnitude of electric field strength [68]. (**b**) Figure showing side view concept sketch of iDEP field constriction system used in fluid integrated circuit (FIC) for cell fusion experiments [36]. Two separate cells are fed into the FIC chamber via a micropump, and once the fusion takes place, the fused cell is transported by fluid pressure to a cell collecting chamber where further post-processing can take place. Figure 3a adapted with Copywrite clearance and reprinted (adapted) with permission from: Copywrite (2008) John Wiley and Sons, ID 4871051145842. ©Figure 3b adapted with Copywrite clearance and reprinted (adapted) with permission from: Copywrite (1989) IEEE, ID 4871050534112.

In the early 1980s, Pohl et al. [68,69] started using the eDEP system (Figure 3a) for cell-to-cell or cell-to-particle fusion and separation experiments in high throughput genetic engineering applications. The experiments resulted in the chain or pearl formation of cells by applying AC electric fields on parallel wire electrodes, eventually leading to cell membrane puncture or damage as cells come into direct contact with the electrodes. To avoid pearl chain formation and cell damage, iDEP technology came into existence. In iDEP, there was no explicit contact between electrodes and cell or media, thereby preventing the generation of toxic species and fouling effects, which makes them ideal for biological applications [45]. The principle of iDEP can be further extended to various low-cost materials, consequently increasing its potential use in high throughput applications. Tracing back to the literature, following Pohl’s work, iDEP was initially reported in 1989 by Masuda et al. [36], who used iDEP to trap and fuse two distinct cells using a pulse electric field with an insulating barrier between electrodes (Figure 3b). Two distinct cell species were fed into a fusion chamber via a micropump and an AC pulse voltage (0–30 V and 2 MHz) on the electrodes to induce a nonuniform electric field in the constriction area at the insulator opening (Figure 3b) [36]. The cells were held at this highest electric field strength constriction region to induce cell fusion. The cell fusion results showed that even in the presence of an insulator barrier between electrodes, a sufficient electric field gradient can be generated. It was also found that electric pulse height and duration influence the fusion time and yield. Masuda et al. [36] further suggested that using precision photolithography machining, higher fusion yields would be obtainable.

Following Masuda′s [36] work, Chou et al. [70] in 2002 used iDEP traps for concentration and patterning of single and double strand DNA. The traps consisted of dielectric constrictions or channels (similar to Masuda’s [36] work) in an insulating material as a substitute of a metallic wire in a conducting ionic buffer solution, thereby generating a local maximum high field gradient [36]. Interestingly, we did not find any major research work on iDEP that was published between Masuda (1989) [36] and Chou′s (2002) reports. The subsequent key development in iDEP was proposed by Cummins and Singh [71] in 2003. Cummins and Singh [71] presented insulating pillar arrays on a glass substrate to manipulate microparticles using DC electric fields. Cummin′s [71] work showed that particle behavior under the electric field can be enhanced by optimizing size and shape of insulating pillars and electrodes. This led to several iDEP based particle separation and characterization reports in the following years.

The use of iDEP in microfluidic devices is becoming increasingly popular compared to conventional DEP based microfluidic platforms. Current iDEP techniques in microfluidic devices are based on two modes of operation: “trapping modes” (Masuda et al. [42] and Shi et al. [72]), for enrichment, and “streaming modes” (Cummins et al. [71]), for particle sorting. Based on these two categories of iDEP modes, advanced iDEP microfluidic devices came into existence, such as, cDEP (contactless DEP) [73,74], curvature induced DEP (c-iDEP)/isomotive DEP (isoDEP) [75,76], and insulator gradient DEP (iGDEP) [77,78]. Davalos et al. [73,74] developed the cDEP mode, wherein the electrodes are positioned on the side walls of the microfluidic channel with insulating posts or pillars running along the length of the channel where the cells are introduced with fluidic pressure. The electrodes are activated to apply fields perpendicular to the fluid flow. Davalos [73,74,79,80] further investigated and characterized cDEP microfluidic device electrode and insulator post geometry to isolate and concentrate cancer cells [73,79,80,81] for rapid point of care separation [67]. Compared to cDEP, c-iDEP based microfluidic devices work on creating nDEP field gradient traps in curved paths/channels. Xu et al. [75] and Allen et al. [76] employed c-iDEP concept in microfluidic devices for particle separation and tracking [76]. Later, Hayes et al. [77] introduced a hybrid iGDEP concept combining both streaming and trapping techniques in a single microfluidic device. iGDEP uses DC fields to stream and trap bioparticles in controlled sawtooth geometric gradients along the length of the microfluidic channel for bioparticle separation applications [77,78]. Past work has shown that all three iDEP approaches (cDEP, c-iDEP, and iGDEP) can be successfully employed in both 2D and 3D microfluidic device platforms. We have presented a brief approach of iDEP implementation in microfluidic systems, but article by Lapizco-Encinas [67] provides a more exhaustive report. Due to the nature of iDEP to operate more gently on particles, the technology continues to gain popularity for the manipulation of sensitive biological systems. In the following sections, we discuss and summarize past and ongoing state-of-art research in detail with regards to iDEP in applications involving cells, viruses, proteins, and nucleic acids.

### 3.1. Cells

Both prokaryotes and eukaryotes, ranging in size from 10 to 120 µm, have been manipulated using iDEP. Recent examples include the work of Suehiro et al. [8], who used an iDEP filter made of spherical glass beads to isolate and manipulate yeast cells, and the work of Lapizco-Encinas et al. [82], in which mixtures of live bacteria (*E. coli*, *B. cereus*, *B. subtilis*, and *B. megaterium*) were separated simultaneously in suspending media. Medically relevant human cell lines have also been manipulated using this technique, with the ultimate goal of achieving high throughput point-of-care disease diagnostics. For example, Chen et al. [83] and Bhattacharya et al. [84] manipulated Hela and breast cancer (MDA-MB-231) mammalian cells, respectively, using iDEP for cancer diagnostics. More recently, Adekanmbi et al. [37] developed a low-cost, high-specificity, iDEP diagnostic tool detecting, concentrating, and separating healthy red blood cells (RBCs) from those infected with the apicomplexan *Babesia*, a haemoprotozoan parasite that is tick-borne. In their device, electrodes with embedded insulated saw-tooth geometry was employed to generate nonuniformity in the electric field. Adekanmbi et. al. [37] were able to separate and isolate *Babesia*-infected RBCs and separate parasites using 6 to 10 V DC on inlet and outlet ports. This novel work demonstrated a portable microfluidic platform for screening donor’s blood for possible babesiosis infections. A list of publications and patents relating iDEP on cells for various applications are further shown in Tables S1 and S2 (Supplementary Materials). While the use of iDEP for cells is well established, as evident by publications, patents, and commercial devices (Charlot Biosciences [31], Sandia National Laboratories [85]), more recent work has employed iDEP to manipulate and separate subcellular biological materials [10,11,18,86].

### 3.2. Viruses and Extracellular Vesicles

Viruses typically vary in size from 20 to 400 nm, and thus they propose challenges in manipulation, as large electrical potentials are typically needed at such small scales. In addition, when the goal of DEP is to detect viruses from clinical samples, low viral loads are common, which adds difficulty in detection [10,87,88]. Several research groups have investigated the effects of eDEP on viruses, experimentally employing metal electrodes [18,66], nanopipettes [49], carbon nanotubes [89], and in droplets [90]. The eDEP based techniques have investigated methods to detect and separate viruses from suspended solutions [50,91,92,93,94,95,96,97,98,99,100,101]. Most work on viruses using eDEP has employed AC rather than DC, as AC the fields can operate at relatively lower voltage regimes, and along with frequency and phase control, aids in limiting electrolysis and electrode damage. Viruses are typically manipulated in eDEP traps using AC fields with peak to peak voltage (V_pp_) between 10 and 20 Vpp and operating frequency ranging from 1 to 50 MHz.

**Figure 4:**
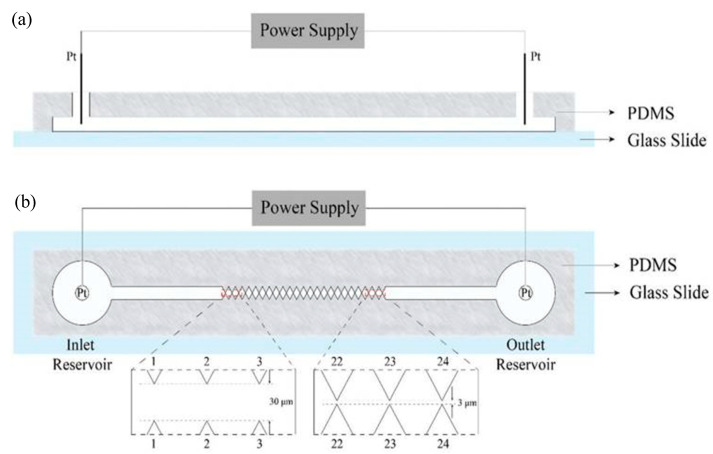
Illustration of the sawtooth g-iDEP device. (**a**) side view and (**b**) top/vertical view. From saw tooth gates 1 to 24, the channel is constricted to an increasing degree by triangular insulting wall protrusions. These constricted zones and structures act as capturing zones due to local increases in electric fields and gradients [103]. ©Figure 4 reproduced with Copywrite clearance and reprinted (adapted) with permission from: Copywrite (1876) Royal Society of Chemistry ID 1044809-1.

Compared to research utilizing eDEP, there are very few reports exploring the behavior of viruses using iDEP devices. In the first reported iDEP-virus investigation, Lapizco-Encinas et al. [102] used iDEP to manipulate the rod-shaped tobacco mosaic virus (TMV) inside a microchannel consisting of an array of insulated posts. The response behavior of TMV particles, such as trapping efficiency was experimentally and numerically investigated. It was found that the TMVs displayed higher trapping thresholds compared to bacterial cells. The iDEP device by Lapizco-Encinas [102] was used to concentrate and remove microbes in water. The next iDEP-virus report came much later, wherein 2014 Masuda et al. [42] reported an active virus filter (AVF) system employing a 3D-constricted flow channel to enable enrichment of influenza viruses. Here, nDEP was used to prevent influenza virus adhesion onto the glass substrate, and then using optical tweezers, the enriched virus was trapped and moved to the desired location to infect selected epithelial lung cells (H292) [42]. It was found that multiple AVFs provided improved efficiency of trapping of the approximately 100 nm diameter viruses. Later, in 2016, Ding et al. [103] demonstrated the enrichment of a smaller virus, *Sindbis* (SVHR), which is approximately 85 nm in diameter, transmitted by mosquitoes, and is the cause of Sindbis fever diseases in humans and animals, whose symptoms include arthralgia, rash, and malaise. In their device, Ding et. al. [103] used photolithography and bonding techniques to fabricate an insulated saw tooth electrode constriction system (4 cm long, with a 500 μm wide, 20 μm open channel) between two reservoirs, as shown in Figure 4. The electrical voltage potential was applied across the entire microchannel (from 0–700 V_pp_) for a minute. After fifteen seconds with 300 V_pp_ applied on the entire channel, the fluorescent-labeled SVHRs accumulated at the smaller saw tooth gate tips (1–3 gates with a 30 µm gap) rather than larger gate tips (22–24 gates with a 3 µm gap). It was found that the viruses accumulated at the gates for a longer time and at a higher V_pp_. Thus, this iDEP device has potential for high-throughput virus manipulation use.

**Figure 5:**
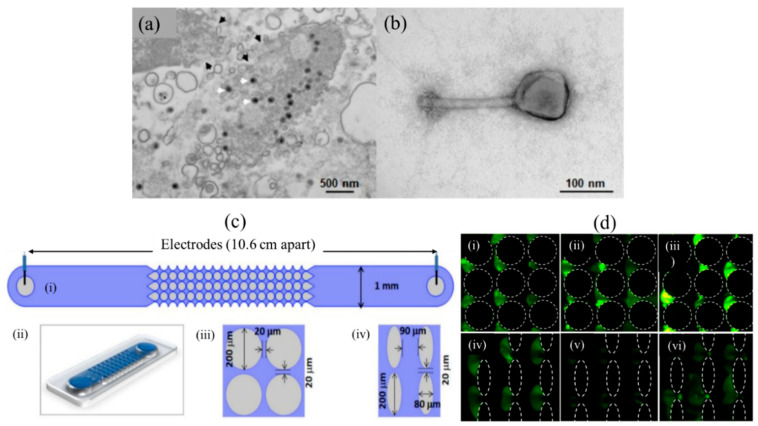
Virion enrichment with iDEP traps [104]. (**a**) Transmission electron microscope (TEM) image of *Salmonella* (SPN3US-infected). The SPN3US progeny particles are indicated with white arrowheads. (**b**) Image of a single SPN3US (Negatively stained) virion showing head (containing dsDNA genome) and tail. (**c**) Sketch and 2D/3D design of iDEP channel. (i): Top view showing schematic of a full channel. (ii): 3D representation of the channel. (iii): Circle diameter 200 µm with gap of 20 µm, (iv): Oval shape posts. (**d**) Circle posts: (i) SPN3US virions at 1200 V, (ii) *P. aeruginosa* phage ϕKZ at 1100 V, and (iii) 1100 V applied on *P. chlororaphis* phage 201ϕ2-1. Oval posts: (iv) SPN3US virion at 800 V, (v) *P. aeruginosa* phage ϕKZ at 750 V and (vi) *P. chlororaphis* phage 201ϕ2-1 at 750 V. [104]. ©Reprinted (adapted) with an open access article distributed under the Creative Commons Attribution License which permits unrestricted use, distribution, and reproduction in any medium.

Currently, the most recent report on iDEP based virus manipulation is the work of Adriana et al. [104] who assessed bacterial virus (phage) separation and enrichment with iDEP traps (Figure 5) on structurally and inherently distinct, phages of *P. chlororaphis* phage 201ϕ2-1, *P. aeruginosa* phage ϕKZ, *S. enterica* phage SPN3US, and it was determined that the virion trapping occurred at lower voltages in devices with circular insulating posts compared to oval-shaped ones (Figure 5d) since these generate lower DEP forces than the latter. The results indicated that virus behavior in the iDEP system could be predicted based on the trapping response of the phage. The trapping voltage for a specific virus (phage) species was found to be reasonably constant using the two distinct insulating post designs, and at the same time, the viability of phages in the applied trapping voltage was maintained. This shows that the trapping voltage can be harnessed to target particular viral species and predict the required voltage for trapping in iDEP devices. Therefore, iDEP is actively being investigated to manipulate various types of viruses and develop innovative high throughput diagnostic devices due to the selective nature of applied trapping voltages on viruses. The findings encountered by Adriana et al. [104] shed light on one such application of iDEP for analyses and separation of phage mixtures. Tables S3 and S4 (Supplementary Materials) further highlight iDEP publications and device patents on various virus manipulation work.

Viruses are closely related to extracellular vesicles (EVs) due to their resemblance in both structural and functional aspects [105]. Unlike viruses, EVs do not replicate and are released by both eukaryotes and prokaryotes; can carry payloads of proteins, lipids, metabolites, and nucleic acids; and play a critical role in cargo transfer and intracellular communication [55,106,107] to facilitate various cellular processes such as cell apoptosis, coagulation, and immune response [56,108,109]. Recently, Sergio et al. [56] created a multisection high throughput microfluidic device to trap and manipulate exosomes using a microfluidic iDEP device which consists of a microchannel having inlet and outlet reservoirs with insulated oval-shaped posts to trap human breast adenocarcinoma (MCF-7) exosomes. High gradient fields of the order of 10^17^ V^2^/m^3^ were generated near the tip of the insulated electrode by applying 200 to 600 V DC in distilled water (conductivity of 14 μS/cm and pH 6.5) containing 2% Tween-20. The experiments at 600 V showed that the dielectrophoretic force produced near the electrode channel caused streaming effects of exosomes, resulting in no trapping. Any voltage between 800 and 1000 V increased flow velocity and damaged the device. It was suggested that exosomes trapped by iDEP may be achievable at higher electric potentials [110]. Following this publication, Sergio et al. [56] in 2019 enhanced the same device and applied 2000 V electric potential difference across the channel length. This time trapping and separation of exosomes in a dielectric electroosmotic flow was achieved in 20 s. These works suggest that iDEP based devices have the potential to be used as an analytical tool to isolate, separate, and investigate subset of exosomes.

**Figure 6:**
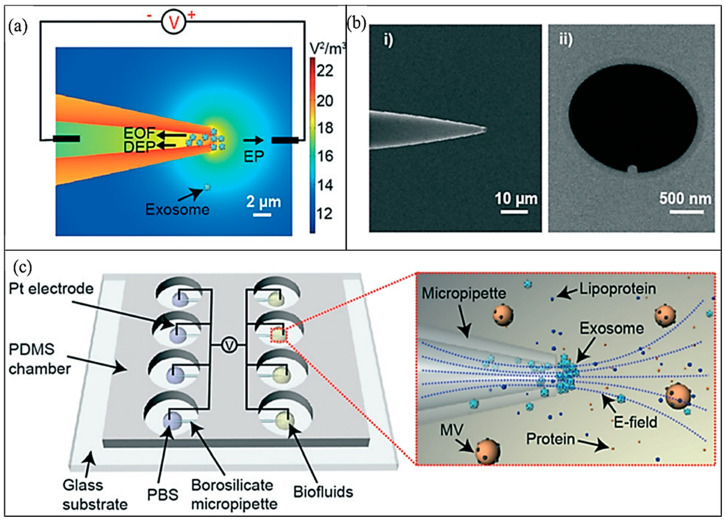
Micropipette iDEP to extract and isolate human plasma exosomes. (**a**) Electric field gradient distribution along with electrokinetic force directions. (**b**) Scanning electron microscopy (SEM) images of pipette made of borosilicate. (i) Pipette side view with angle, (ii) pipette′s tip top view. (**c**) Illustration of micro device with an array of 4 parallel micropipettes in a Polydimethylsiloxane (PDMS) chamber [72]. ©Figure 6 reproduced with Copywrite clearance and reprinted (adapted) with permission from: Copywrite (2001) Royal Society of Chemistry, ID 1039126-1.

In earlier work on iDEP of exosomes, microfluidic devices employed larger quantities (milliliter order) of distilled water as the suspended media or flow media; however, clinical liquid biopsy sample biofluids containing exosomes still require laborious steps to purify and concentrate sufficient working volumes [111]. To overcome this barrier, Shi et al. [72] in 2019 presented a micropipette integrated iDEP technology for the rapid isolation and trapping of human plasma exosomes from biofluids and conditioned cell culture media. A small sample volume of 200 μL media was used to isolate and separate exosomes within 20 min under a relatively low DC field of the order of 10 V/cm. The micro device is made of a glass micropipette (Figure 6) along with two platinum electrodes: one in the conical pipette and another touching the sample of 200 μL volume. The pipette conical geometry of the pore along with the electrode’s activation produces a strong DEP force in the vicinity of the pipette′s tip region [72]. The DEP force is balanced by electroosmosis and electrophoresis forces creating a suitable trapping zone. The work of Shi et al. [72] not only manipulated exosomes in the unique microsystem, but he also calibrated the effect of exosomes trapping in different media volumes such as a serum, plasma, and saliva. The superior yield of isolated exosomes in the device was confirmed by the Enzyme-Linked Immunosorbent Assay (ELISA) quantification [72]. The number of exosomes extracted in the pipette was quantified in different media extraction. The quantified results showed increased concentrations of isolated nanovesicles using iDEP compared to the conventional differential centrifugation (DU) technique. Shi et al. [72] extended the work to a parallel array of micropipettes as shown in Figure 6c. Nonetheless, their work provided a kit to trap and isolate the exosomes avoiding conventional photolithography techniques to produce microelectrodes and provided an insight into the quantification techniques for analyzing exosome counts.

### 3.3. Proteins

**Figure 7:**
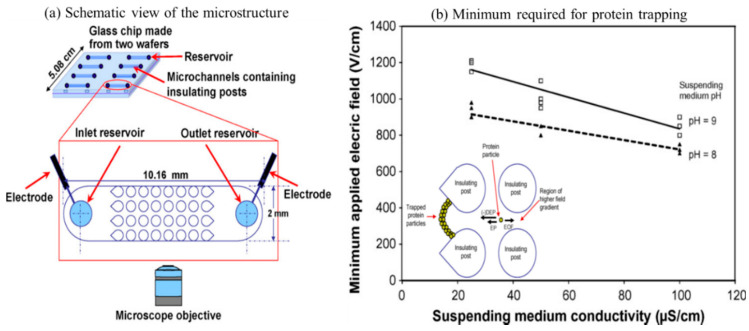
(**a**) Schematic representation of microchip to manipulate proteins. DC electric field applied across an array of insulating posts [112]. (**b**) Plot shows the protein trapping trend in a varying suspending media conductivity and pH vs. minimum applied field strength [112]. The trapped protein particles are shown to experience EP and i-DEP within the region of high field gradient. ©Figure 7 adapted with Copywrite clearance and reprinted (adapted) with permission from: Copywrite (2008) Elsevier ID 4844020262896.

The first work of employing iDEP to manipulate proteins was reported by Lapizco-Encinas et al. [112] in 2008. In their work, bovine serum albumin proteins were fluorescently labeled and concentrated inside of a glass cylindrical insulating microstructure array (Figure 7a). Different media conductivity (25, 50, and 100 S/cm) was used to measure the function of the conductivity of particle suspending media in a DC field. The applied field strength of 700–1600 V/cm and suspending medium properties were shown to induce stronger DEP response of the proteins. Moreover, it was shown that when higher DC fields were employed in low conductivity suspending medium, the proteins experienced nDEP and effective trapping occurs (Figure 7b) [112]. The findings by Lapizco-Encinas et al. [112] showed necessary operable conditions of microdevices for iDEP with applications for the bioparticle concentration [112]. Based on his electrode design guidelines, research on iDEP based proteins manipulation era started [24,78,112,113,114], such as that reported by Nakano et al. [24,115], where 3D insulated pillar electrodes were developed to manipulate the immunoglobulin G (IgG) antibody.

**Figure 8:**
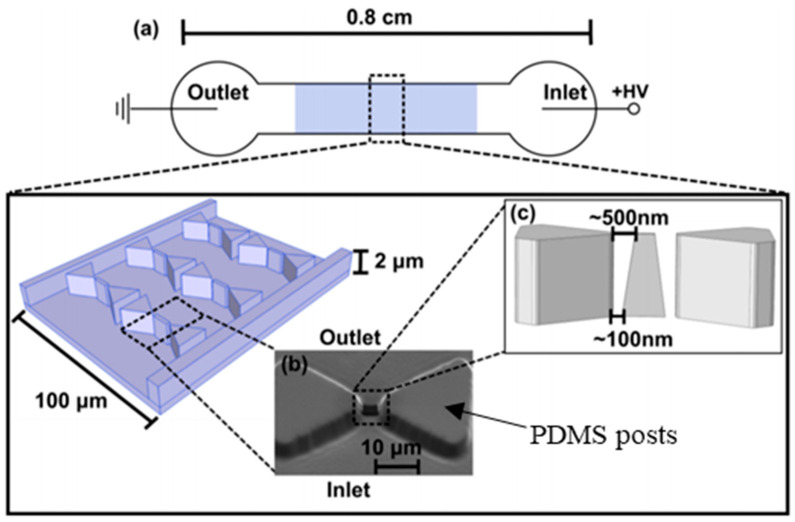
Schematic top view of the iDEP micro device (not to scale) [116]. (**a**) An electric potential difference applied across microchannel inlet and outlet showing insulated the array of posts at the center of the device. (**b**) A tiny nanoconstriction outlet where nm-size proteins are manipulated between the tips of triangular microposts. (**c**) Image showing triangular post dimensions along with gap between nanoconstrictions in the PDMS mold [116]. ©Figure 8 adapted with Copywrite Clearance and reprinted (adapted) with permission from: Copywrite (2001) Royal Society of Chemistry, ID 1048996-1.

Rapid separation techniques based on iDEP are particularly attractive for protein manipulation in particularly intricate fluid combinations such as biofluids or cell lysates. However, the process of DEP transport of protein polarization is not well understood. In 2015, Nakano et al. [116] improved on earlier IgG work [24] where they created a novel 3D nonconstriction iDEP device (Figure 8) to show polarization of protein β-galactosidase in nDEP under DC conditions. Numerical and experimental work on the effect of electrokinetic motion of proteins on the extent of DEP mobility was investigated. It was observed that unique voltage dependent ion and β-galactosidase concentrations influenced protein concentration at device nano constrictions [116]. Nakano’s work provides an understanding of the factors influencing protein transport in iDEP, which is essential for protein preconcentration and separation. Their research indicates the potential for using 3D electrode-based iDEP devices to manipulate proteins, compared to traditional 2D electrodes. While there is an increasing amount of curiosity in iDEP based protein manipulation, there is an ”n” number of proteins, and the response of each protein type to the applied iDEP may be different. It is also extremely challenging to produce high enough electric field gradients to generate sufficiently large DEP forces to compete with electrothermal and molecular diffusion forces [116]. Moreover, low sample volume raises analytical challenges. The assays developed by Lapizco-Encinas et. al. [112] and Nakamo et. al. [116] help to overcome these challenges and provide an insight into the understanding of complex protein structure interactions with electrokinetic fields. For future advancements in iDEP protein-based assays, the mechanism of protein polarization must be investigated further as it plays a vital role in protein separation and screening for therapeutic purposes.

### 3.4. Nucleic Acids

Reports on iDEP based DNA molecule manipulation surfaced in 2002 when in seminal work by Asbury et al. [117] were a microfluidic device used to evaluate two proposed methods for measuring the efficiency of DEP traps. In the first method, DNA molecules were isolated and trapped at the edges of gold-film electrodes and peak fluorescence levels of trapped DNA molecules were measured. After 30 s, the electric field was switched off and the peak fluorescence levels of dispersed DNA molecules were recorded. After 30 s, the fluorescence wide peaks shortened trapping efficiency due to the dispersion of smaller DNA molecules. Fluorescence peak height distributions indicated a slight tendency for larger molecules to be more easily trapped. In the second method, fluorescence intensity vs. time was measured at a varying AC frequency. Measuring the number of trapped DNA, the peak height was determined. Asbury et al. [117] further calibrated frequency and optimum voltage required to trap the DNA molecules. In the article, it was clearly mentioned that the manipulation of small amounts of DNA by electrical forces is a likely technique to grow in the near future. In 2004, Ying et al., [118] developed a nanopipette system to manipulate and trap DNA and single nucleotide in low frequency AC fields. Later in 2007, Regtmeier et al. [119] developed an iDEP microfluidic device to separate a linear DNA (λ (48.5 kbp), circular plasmid DNA (7 and 14 kbp), and T2 (164 kbp) DNA) by electrophoresis [119]. Regtmeier [119] proposed that the electrophoretic force is responsive to several DNA fragments because of length-dependent DNA polarizabilities. He further attributed that the same microfluidic device can be further used to separate proteins and biomolecules [119]. In 2009, Swami et al. [120] demonstrated a constriction-based DEP within a microfluidic channel to enhance the focusing effects of the constrictions and edges. In these constrictions, it was shown that the preconcentration of target DNA can be immobilized. This resulted in improved (up to ten-fold) hybridization kinetics of DNA at target concentration values [120].

**Figure 9:**
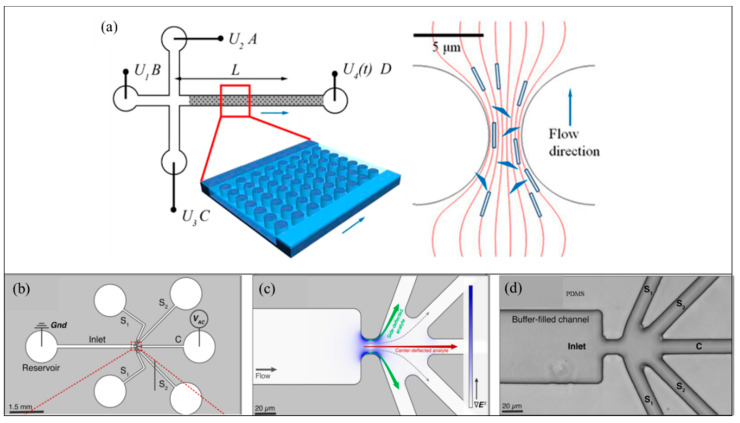
DNA manipulation using iDEP [121]. (**a**) Sketch of the microfluidic device (not to scale). Channels linked to reservoirs (A−C) are 1.7 cm long. All the channels have a width of 100 μm and a depth of 10 μm. The length between the post arrays linking to reservoir D along the channel is 2 cm. The distance between the intersection and detection point is L. [121]. (**b**) Schematic to show the pathways for introducing sample DNA analyte via the left reservoir. From the left reservoir, the analyte enters the constriction via inlet branch and then electric potential is applied to five outlet branches (S1 or S2), and C. Inlet and C outlet reservoirs are inserted with platinum wires [121]. (**c**) Zoomed in view of the constriction region showing the intensity of electric field gradient color. (**d**) Bright-field image of the expanded region of the constriction zone in the microdevice [121]. © Figure 9 Reprinted and adapted with permission from [121]. Copyright (2015) American Chemical Society.

Later in 2015, Gan et al. [121] manipulated origami DNA in a microdevice using the iDEP technique and measured the polarizability of the origami species (Figure 9a). Polarizability of the origami DNA was found to be responsible for the DNA migration in the applied iDEP field [121]. Six-helix bundle origami (6HxB) polarizabilities were experimentally investigated, and triangle origami [121] were reported. The experiments further discuss origami species orientation influence with respect to diffusion escape process in the dielectrophoretic trap. In 2017, Jones et al. [122] tailored a microfluidic sorter to separate various DNA analytes into separate channel outlets, as shown in Figure 9c,d. The 1.0, 10.2, 19.5, and 48.5 kbp dsDNA analytes, including both genomic DNA and plasmid that are selectively separated by optimized AC electric fields [122]. The analytes were introduced through the circular reservoir (Figure 9a). Then, the analytes were transported to the constriction via the inlet reservoir and then to the five outlet branches. The inlet and outlet ”C” reservoir are inserted with platinum wires onto which electric potential was applied. The iDEP forces selectively (by a suitable frequency and amplitude) redirect analyte from original flow paths and collected in the C or side outlets. This selective deflection of DNA is used in fractionation for a range of downstream analysis applications.

**Figure 10:**
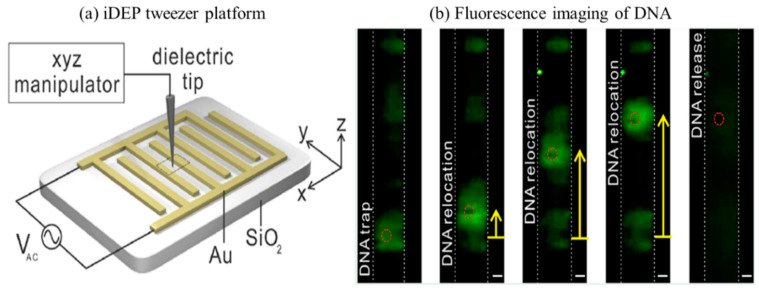
iDEP manipulation with optical tweezer [123]. (**a**) Graphic diagram of the device showing an insulating tip with xyz manipulator control. (**b**) Image shows fluorescence-labeled DNA being trapped, relocated, and released spatially with iDEP tweezers. The double-stranded λ-DNA fragments were green fluorescent labeled with YOYO-1 trap DNA. An AC voltage (7 V, 200 kHz) applied to manipulate DNA. All scale bars are 5 μm [123]. ©Figure 10 Reprinted (adapted) with permission from [123]. Copyright (2020) American Chemical Society.

In early 2020, Oh et al. [123] reported an iDEP tweezers platform to manipulate and selectively separate DNA and polystyrene particles. The strength and polarity of the tweezers were determined numerically and experimentally. Figure 10a shows the arrangement of interdigitated gold (Au) electrode array patterned on Silicon dioxide substrate. The insulator dielectric tip was made of glass rod and moved with an ”xyz” manipulator whilst trapping, translating, and releasing biomolecules. External AC voltage was applied on the electrodes and with the help of a fluorescence microscope, DNA manipulation was captured, as shown in Figure 10b. The low-frequency AC voltage seems to attract DNA to the electrode (at 9 V, 80 kHz) generating a strong pDEP effect, whereas DNA was found to experience slightly reduced pDEP at the increased frequency of 120 kHz, which triggers the confined DNA to slightly move away from the electrode [123]. At 200 kHz and the same voltage of 9 V, the DNA was entirely stretched, suggesting a considerable dependence of the DEP frequency of the applied AC voltage. The results indicated that the strength of iDEP dominant over thermal force for DNA manipulation. The technique effectively separated DNA in a fluid microfluidic environment without fouling, electrolysis, and joule heating [123].

## 4. Knowledge Gaps and Future Directions

There is an enormous interest in manipulating biomolecules, individually and as populations, for both scientific and clinical diagnostic applications. The smaller the size of these biomolecules, the greater the challenge to manipulate with a certain degree of control on the viability. The following are some of the key knowledge gaps that are being actively investigated but need further attention in the future.

### 4.1. Overcoming Effects of High Electric Field and Gradients

**Figure 11:**
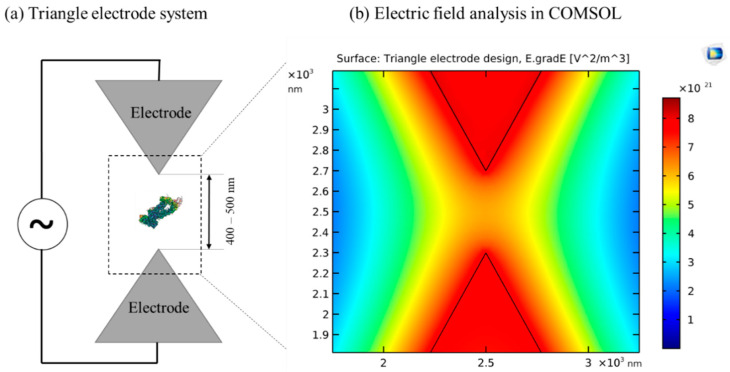
Illustration of triangular electrodes used by Hölzel [124] to manipulate proteins. (**a**) Sketch of triangular electrodes with 500 nm spacing provided between two electrodes to trap proteins with the help of a suitable electric potential. (**b**) Analysis of electric field and gradient in COMSOL v4.0 AC/DC module. The color intensity map shows the electrode plane field gradient. Near the center of the electrodes, there is a very high gradient generated of the order of ≈5.5 × 10^21^ V^^2^/M^^3^. ©Figure 11a adapted with Copywrite clearance and reprinted (adapted) with permission from: Copyright (2020) American Physical Society, ID: RNP/20/JUL/028426.

To manipulate a single molecule, such as a protein, a typical DEP device requires a field gradient of 10^21^ V^2^/m^3^ [33,45,124]. Optimum field gradients as shown by R. Hölzel et al. [124] can be obtained by calibrating specific criteria, such as 500 nm spacing between electrodes, with a 10 V electrical signal (Figure 11). At this electric field strength, toxic species can be generated if electrodes are not properly insulated or shielded. Insulator based DEP could be used to overcome this issue by eliminating the direct contact of electrodes from the media. However, generating 10^21^ V^2^/m^3^ field gradients using iDEP is a challenge, since the limiting factor here is the insulator barrier which significantly reduces the field gradient. Therefore, to overcome the electric field gradient issue, future materials research is necessary to develop advanced insulators and electrodes capable of producing higher maximum gradient fields with low heat and minimal or no toxic species generation. For this purpose, a silicon-based iDEP device was reported by P. Zellner et al. [125] to apply minimized heating effects on the biological specimens in extremely dilute solutions for next-generation sorting and concentration. The silicon device was shown to minimize the heating effects, but the fabrication process increased complexity as specified by the author. Single-walled carbon nanotubes (SWNTs) were explored for iDEP manipulation of single-stranded DNA-wrapped SWNTs [126]. SWNTs have unique structural, mechanical, and optical properties that can be utilized to enhance field gradients. More recently, a silicon-based 3D passivated electrode [127] was developed to produce higher gradient fields at low applied voltage. These advanced material technologies provide advancements to overcome the electric gradient issues relating to iDEP; however, implementing such novel materials in micro/nano scale and fabricating the devices remains an area of unexplored research.

### 4.2. Optimization of Fluid Micro-Environments

Medium conductivity and its pH are two major parameters influencing cell viability in all DEP devices. More specifically, the ability to handle highly conductive suspension media at physiological pHs (7–8) with negligible joule heating is necessary for biological samples. While the conductivity of the fluid micro-environment can be easily varied via the gradient field, this results in increasing joule heat. Joule heating is directly related to pH changes, and, therefore, heating of media needs to be avoided during any cell manipulation experiments. During each experiment, variables such as media conductivity, insulator/electrode material, and applied frequency and amplitude, need to be optimized and calibrated to achieve the desired throughput. A systematic method for optimizing these variables and predicting the joule heating in the fluid environment will, therefore, significantly advance DEP research. Such optimization and prediction can be performed by a suitable machine learning algorithm. Recently, machine learning has been used in cell detection, biosensing, and predicting cell dielectric spectrum parameters [128,129]. Lannin et al. [128,130] used a machine learning algorithm to locate and classify thousands of cells via imaging data and a Matlab machine learning toolbox. Lannin [128,130] further measured electrical properties of cancer and algae cells such as cross over frequency, membrane capacitance, and cytoplasm conductivity using DEP and electrorotation experiments and then used the experimental data to predict and optimize dielectric spectra using machine learning algorithm. Once the machine learning algorithm builds predicted data of dielectric spectra, one can then backtrack the DEP device parameters. Similarly, Honrado et al. [131] used machine learning to evaluate and record electrical fingerprints for the accurate detection of thousands of red blood cells and yeasts [131]. The work by Lannin and Honrado [128,130,131] shows that machine learning can be employed in the iDEP device as well, since the only integration with the device is the image based analysis and data management with the predictable machine learning algorithms. We believe that in the near future, integrating machine learning with real-time data analysis in a DEP device will further enhance our understanding of fluid micro-environments, enabling the optimized manipulation of biological specimens and prediction of sample parameters, such as polarization rate of proteins and dielectric spectra of cells.

## 5. Conclusions

The iDEP technique has advanced significantly over the last several years, especially with respect to the manipulation of biological material. Current research on devices using iDEP have demonstrated that the method can be sensitive enough to manipulate single molecules, while also being gentle enough to maintain biomolecule functionality. A major advantage of iDEP is its ability to limit Joule fluid heating, due to the presence of an insulating barrier between the electrode and media. This aids in maintaining cell viability and biological particle integrity compared to conventional methods. Along with the benefit of maintaining cellular integrity, the safe and optimized electrical energy used in iDEP can induce the cellular migration of certain immune cells. Therefore, iDEP has direct applications outside of single particles and can be potentially applied in wound healing, tissue repair, biomedicine, and targeted delivery applications [132,133]. Certain immune cells are able to be activated with small electric fields and gradients to jump start wound healing to reduce infection [132]. In addition, with an optimum iDEP electrode and insulator geometry, calibrated electric field gradients can be generated in which a single cell in a microchannel can be selectively positioned allowing highly efficient transfection for applications in targeted delivery and biomedicine. Expected future integration of iDEP with super resolution imaging and 3D microfluidic traps has tremendous potential in manipulating particles for protein folding, blood and drug screening, separations, and molecular sensing applications. Insulator-based DEP technologies are being driven primarily by biomolecular and biomedical applications, especially at the single molecule scale. Recent advances indicate that the method has the potential to be widely used in the future in high throughput scientific and clinical devices.

## Figures and Tables

**Table 1:** Known Clausius–Mossotti (CM) factor equations employed in micro, nano, and molecular regime.

	Type of Cells or Particles	Formula
Micro Scale	Solid Sphere [44]	$\frac{{\tilde{\epsilon }}_{p}{}^{*}-{\tilde{\epsilon }}_{m}{}^{*}}{{\tilde{\epsilon }}_{p}{}^{*}+2{\tilde{\epsilon }}_{m}{}^{*}}$
	Two Shell ellipsoid [52]	$\frac{{\tilde{\epsilon }}_{p}{}^{*}-{\tilde{\epsilon }}_{m}{}^{*}}{3{\tilde{\epsilon }}_{m}{}^{*}+A_{i}\left({\tilde{\epsilon }}_{p}{}^{*}-{\tilde{\epsilon }}_{m}{}^{*}\right)}$
	Typical cell (single shell) [53]	${\tilde{\epsilon }}_{\mathrm{cm}}{}^{*}\frac{{\left(\frac{r_{ext}}{r_{ext}-th_{cm}}\right)}^{3}+2\left(\frac{{\tilde{\epsilon }}_{cp}{}^{*}-{\tilde{\epsilon }}_{cm}{}^{*}}{{\tilde{\epsilon }}_{cp}{}^{*}+2{\tilde{\epsilon }}_{cm}{}^{*}}\right)}{{\left(\frac{r_{ext}}{r_{ext}-th_{cm}}\right)}^{3}-\left(\frac{{\tilde{\epsilon }}_{cp}{}^{*}-{\tilde{\epsilon }}_{cm}{}^{*}}{{\tilde{\epsilon }}_{cp}{}^{*}+2{\tilde{\epsilon }}_{cm}{}^{*}}\right)}$
Submicro/Nano Scale	Bacteria [45,52,53] Virus [50,54] Exosomes [55,56]	$\frac{{\tilde{\epsilon }}_{p}{}^{*}-{\tilde{\epsilon }}_{m}{}^{*}}{{\tilde{\epsilon }}_{p}{}^{*}+2{\tilde{\epsilon }}_{m}{}^{*}}$ or ${\tilde{\epsilon }}_{cm}{}^{*}\frac{{\left(\frac{r_{ext}}{r_{ext}-th_{cm}}\right)}^{3}+2\left(\frac{{\tilde{\epsilon }}_{cp}{}^{*}-{\tilde{\epsilon }}_{cm}{}^{*}}{{\tilde{\epsilon }}_{cp}{}^{*}+2{\tilde{\epsilon }}_{cm}{}^{*}}\right)}{{\left(\frac{r_{ext}}{r_{ext}-th_{cm}}\right)}^{3}-\left(\frac{{\tilde{\epsilon }}_{cp}{}^{*}-{\tilde{\epsilon }}_{cm}{}^{*}}{{\tilde{\epsilon }}_{cp}{}^{*}+2{\tilde{\epsilon }}_{cm}{}^{*}}\right)}$
Molecular Scale	Proteins [45] DNA [45] Biomolecules [45]	$\left(\frac{\epsilon_{m}-1}{\epsilon_{m}+2}\right)\frac{1}{\rho }=\frac{N_{A}\alpha }{3M\epsilon_{0}}$

* $A_{i}$ is a depolarization component factor along ellipsoid axis (i = x, y, z); * $r_{ext}$—external radius of cell; $th_{cm}$—thickness of cell membrane; ${\tilde{\epsilon }}_{\mathrm{cp}}$—cell cytoplasm complex permittivity; * ${\tilde{\epsilon }}_{\mathrm{cm}}$—cell membrane complex permittivity.

## Supplementary Materials

The following are available online at https://www.mdpi.com/1424-8220/20/18/5095/s1, Table S1: iDEP/cDEP investigations of cells., Table S2: Patents on iDEP manipulation of cells, Table S3: iDEP investigations on virus manipulation, and Table S4: Patents on iDEP manipulation of viruses.
