# Supplementary material for: Insulator Based Dielectrophoresis: Micro, Nano, and Molecular Scale Biological Applications
Source: Sensors (Basel). 2020 Sep 7;20(18):5095. doi: 10.3390/s20185095 (PMC7570478; doi:10.3390/s20185095)
Supplement: Supplementary file 1 [file sensors-20-05095-s001.pdf]

sensors

MDPI

Supplementary Materials

Insulator Based Dielectrophoresis: Micro, Nano, and Molecular Scale Biological Applications

Prateek Benhal [1,2,*], David Quashie [1,2], Yoontae Kim [3] and Jamel Ali [1,2,*]

[1] Department of Chemical and Biomedical Engineering, FAMU-FSU College of Engineering, Tallahassee, FL 32310, USA; david1.quashie@famu.edu
[2] National High Magnetic Field Laboratory, Tallahassee, FL 32310, USA
[3] American Dental Association Research & Science Institute, Gaithersburg, MD 20899, USA; kimyo@ada.org
* Correspondence: pbenhal@eng.famu.fsu.edu (P.B.); jali@eng.famu.fsu.edu (J.A.); Tel.: +1-850-410-6282 (J.A.)

Table S1. iDEP/cDEP investigations of cells.

Cell type	Cell size (μm)	Electrode material	Suspending media	Type of electric field (DC)	Application	Ref.
Bacteria: *E.coli*, *S. mitis*, and *S. aureus*	2	Platinum electrodes	Bovine serum albumin (BSA)/cell suspension media	0–3000 V_{pp}	Selective and controlled manipulation	[1–7]
Bacteria: *Megaterium*	4	platinum-wire electrodes	DI water	40 V_{pp}	Selective and controlled manipulation	[8]
Yeast	3–4	platinum-wire electrodes	0.5 mmol/L KH_2PO_4 in DI water	600–1200 V_{pp}	Selective and controlled manipulation	[9–12]
Bacteria: *C. crescentus*	3–4	platinum-wire electrodes	DI water	40 V_{pp}	Selective and controlled manipulation	[8,13]
Bacteria: *B. subtilis*	4–10	platinum-wire electrodes	DI water and Luria-Bertani (LB) nutrient broth	30 V_{pp}	Trapping, concentration, and separation	[14]
Cancer cells: MDA-MB-231, MCF 7, MOSE, CTCs	~10-20	platinum-wire electrodes	HEPES cell buffer media	~0–270 V_{pp}	Selective trapping, detection, separation, enrichment, and manipulation	[15–22]
Erythrocytes	6–8	Platinum electrodes	Dextrose in PBS	17.1–68.5 V_{pp}	Selective trapping, detection, and separation	[23]
HeLa	~40	Gold	Sucrose in PBS	2.83×10^4 V_{pp} /m	Selective trapping, detection, and separation	[24,25]
Sperm/Egg cells	~100–120	Platinum-wire electrodes	HEPES buffer medium	200–1500 V_{pp}	Enrichment, manipulation, and rapid purification	[26]

Neurons	3–18	Platinum electrodes	5 g/100mL (%) BSA (bovine serum album) in PBS	90 V_{pp}	Selective trapping, and characterizition	[27,28]
Microalgae	40–150	Platinum-wire electrodes	Bidistilled water and Modified Bold 3 N medium, pH 6.2	100–1000 V_{pp}	Concentration and seperation	[29]
Liposomes, mitochondria	0.3	Platinum electrodes	1 mM F108, 10 mM HEPES, pH adjusted to 7.4 by KOH	10–800 V_{pp}	Separation	[30]
RBCs	7.5 to 8.7	Platinum electrodes	Diluted blood samples	50 V_{pp}	Trapping and real time monitoring	[31]

Table S2. Patents on iDEP manipulation of cells

Patent title	Application	Patent Number	References
Devices and methods for contactless dielectrophoresis for cell or particle manipulation, Davalos Rafael V. et al., 2019	Manipulation and sorting	US 20190137446	[32]
Identification and monitoring of cells by dielectrophoretic tracking of electrophysiology and phenotype, Swami Nathan et al., 2017	Isolation, detection, and trapping	US 20170218424 A	[33]
Insulator-based DEP with impedance measurements for analyte detection, Davalos Rafael V. et al., 2017	Analysis and impedance detection	US 7678256 B2	[34]
Insulator-based DEP with impedance measurements for analyte detection, Davalos Rafael V. et al., 2008	Detection and analysis	US 20080105565 A1	[35]
Dielectrophoresis device and method having nonuniform arrays for manipulating particles, Cummings Eric B. et al., 2008	Manipulation and detection	US 7419574	[36]

Table S3. iDEP investigations on virus manipulation

Type of virus	Size (nm)	Electrode material	Suspended media	Type of electric field	Application	References
Influenza virus (A PR/8)	80–120	Gold and chromium	HEPES and KCl	10 MHz, 20 V_{pp} AC	Selective and controlled manipulation, enrichment	[37]
Bacteriophage	24–200	Platinum wire	LB agar and sterilized DI water	750–800 V_{pp} AC	High throughput manipulation	[38]
Tobacco mosaic virus	18–300	Platinum wire electrodes	Carbonate buffer	2000 V_{pp}/cm DC	Force response	[39]
Sindbis Virus	~21.16	Platinum wire	NaCl and PIPES	0–700 V_{pp} DC	Controlled trapping	[40]

Table S4. Patents on iDEP manipulation of viruses

Patent title	Application	Patent number	References
Devices and methods for contactless dielectrophoresis for cell or particle manipulation, Davalos et al., 2015	Cell and virus manipulation	US 8968542 B2	[41]
System for particle concentration and detection, United States Patent, Morales et al., 2013	Collection of airborne virus pathogens	US 8398839 B1	[42]
Method for concentration and separation of biological organisms by ultrafiltration and dielectrophoresis, Simmons, et al., 2012	Detection and separation	US 8257568 B1	[43]
Insulator-Based DEP with Impedance Measurements for Analyte Detection, Davalos Rafael V. et al., 2017	Analyte detection and separation	US 7678256 B2	[44]
Dielectrophoresis device and method having insulating ridges for manipulating particles, Cummings, et al., 2008	Concentration, separation, and capture	US 7347923 B2	[45]
Dielectrophoretic systems without embedded electrodes, Cummings, et al., 2004	Separation	US 20040026250 A1	[46]
Electrodeless dielectrophoresis for polarizable particles, Robert et al., 2001	Virus separation	WO2001037958A2	[47]

References

1. Jones, P.V.; DeMichele, A.F.; Kemp, L.; Hayes, M.A. Differentiation of Escherichia coli serotypes using DC gradient insulator dielectrophoresis. *Analytical and bioanalytical chemistry* **2014**; Vol. *406*, pp 183-192, doi:10.1007/s00216-013-7437-5.
2. Zellner, P.; Shake, T.; Sahari, A.; Behkam, B.; Agah, M. Off-chip passivated-electrode, insulator-based dielectrophoresis (OπDEP). *Analytical and bioanalytical chemistry* **2013**; Vol. *405*, pp 6657-6666, doi:10.1007/s00216-013-7123-7.

3. Nakidde, D.; Zellner, P.; Alemi, M.M.; Shake, T.; Hosseini, Y.; Riquelme, M.V.; Pruden, A.; Agah, M. Three dimensional passivated-electrode insulator-based dielectrophoresis. *Biomicrofluidics* **2015**; Vol. *9*, pp 014125, doi:10.1063/1.4913497.
4. Pysher, M.D.; Hayes, M.A. Electrophoretic and dielectrophoretic field gradient technique for separating bioparticles. *Anal Chem* **2007**; Vol. *79*, pp 4552-4557, doi:10.1021/ac070534j.
5. Braff, W.A.; Willner, D.; Hugenholtz, P.; Rabaey, K.; Buie, C.R. Dielectrophoresis-based discrimination of bacteria at the strain level based on their surface properties. *PLoS One* **2013**; Vol. *8*, pp e76751-e76751, doi:10.1371/journal.pone.0076751.
6. Braff, W.A.; Pignier, A.; Buie, C.R. High sensitivity three-dimensional insulator-based dielectrophoresis. *Lab on a Chip* **2012**; Vol. *12*, pp 1327-1331, doi:10.1039/C2LC21212A.
7. Cho, Y.-K.; Kim, S.; Lee, K.; Park, C.; Lee, J.-G.; Ko, C. Bacteria concentration using a membrane type insulator-based dielectrophoresis in a plastic chip. *Electrophoresis* **2009**; Vol. *30*, pp 3153-3159, doi:10.1002/elps.200900179.
8. Lapizco-Encinas, B.H.; Simmons, B.A.; Cummings, E.B.; Fintschenko, Y. Insulator-based dielectrophoresis for the selective concentration and separation of live bacteria in water. *Electrophoresis* **2004**; Vol. *25*, pp 1695-1704, doi:10.1002/elps.200405899.
9. Moncada-Hernandez, H.; Baylon-Cardiel, J.L.; Pérez-González, V.H.; Lapizco-Encinas, B.H. Insulator-based dielectrophoresis of microorganisms: Theoretical and experimental results. *Electrophoresis* **2011**; Vol. *32*, pp 2502-2511, doi:10.1002/elps.201100168.
10. Kang, Y.; Cetin, B.; Wu, Z.; Li, D. Continuous particle separation with localized AC-dielectrophoresis using embedded electrodes and an insulating hurdle. *Electrochimica Acta* **2009**; Vol. *54*, pp 1715-1720, doi:https://doi.org/10.1016/j.electacta.2008.09.062.
11. Çetin, B.; Li, D. Lab-on-a-chip device for continuous particle and cell separation based on electrical properties via alternating current dielectrophoresis. *Electrophoresis* **2010**; Vol. *31*, pp 3035-3043, doi:10.1002/elps.201000107.
12. Lewpiriyawong, N.; Xu, G.; Yang, C. Enhanced cell trapping throughput using DC-biased AC electric field in a dielectrophoresis-based fluidic device with densely packed silica beads. *Electrophoresis* **2018**; Vol. *39*, pp 878-886, doi:10.1002/elps.201700395.
13. Kovarik, M.L.; Jacobson, S.C. Integrated Nanopore/Microchannel Devices for ac Electrokinetic Trapping of Particles. *Analytical Chemistry* **2008**; Vol. *80*, pp 657-664, doi:10.1021/ac701759f.
14. Barrett, L.M.; Skulan, A.J.; Singh, A.K.; Cummings, E.B.; Fiechtner, G.J. Dielectrophoretic Manipulation of Particles and Cells Using Insulating Ridges in Faceted Prism Microchannels. *Analytical Chemistry* **2005**; Vol. *77*, pp 6798-6804, doi:10.1021/ac0507791.
15. Bhattacharya, S.; Chao, T.C.; Ros, A. Insulator-based dielectrophoretic single particle and single cancer cell trapping. *Electrophoresis* **2011**; Vol. *32*, pp 2550-2558, doi:10.1002/elps.201100066.
16. Kang, Y.; Li, D.; Kalams, S.A.; Eid, J.E. DC-Dielectrophoretic separation of biological cells by size. *Biomedical microdevices* **2008**; Vol. *10*, pp 243-249, doi:10.1007/s10544-007-9130-y.
17. Soltanian-Zadeh, S.; Kikkeri, K.; Shajahan-Haq, A.N.; Strobl, J.; Clarke, R.; Agah, M. Breast cancer cell obatoclax response characterization using passivated-electrode insulator-based dielectrophoresis. *Electrophoresis* **2017**; Vol. *38*, pp 1988-1995, doi:10.1002/elps.201600447.
18. Shafiee, H.; Caldwell, J.L.; Sano, M.B.; Davalos, R.V. Contactless dielectrophoresis: a new technique for cell manipulation. *Biomedical microdevices* **2009**; Vol. *11*, pp 997, doi:10.1007/s10544-009-9317-5.

19. Douglas, T.A.; Cemazar, J.; Balani, N.; Sweeney, D.C.; Schmelz, E.M.; Davalos, R.V. A feasibility study for enrichment of highly aggressive cancer subpopulations by their biophysical properties via dielectrophoresis enhanced with synergistic fluid flow. *Electrophoresis* **2017**; Vol. *38*, pp 1507-1514, doi:10.1002/elps.201600530.
20. Elvington, E.S.; Salmanzadeh, A.; Stremler, M.A.; Davalos, R.V. Label-free isolation and enrichment of cells through contactless dielectrophoresis. *J Vis Exp* **2013**; 10.3791/50634, pp, doi:10.3791/50634.
21. Salmanzadeh, A.; Kittur, H.; Sano, M.B.; C Roberts, P.; Schmelz, E.M.; Davalos, R.V. Dielectrophoretic differentiation of mouse ovarian surface epithelial cells, macrophages, and fibroblasts using contactless dielectrophoresis. *Biomicrofluidics* **2012**; Vol. *6*, pp 24104-2410413, doi:10.1063/1.3699973.
22. Aghilinejad, A.; Aghaamoo, M.; Chen, X.; Xu, J. Effects of electrothermal vortices on insulator-based dielectrophoresis for circulating tumor cell separation. *Electrophoresis* **2018**; Vol. *39*, pp 869-877, doi:10.1002/elps.201700264.
23. Srivastava, S.K.; Artemiou, A.; Minerick, A.R. Direct current insulator-based dielectrophoretic characterization of erythrocytes: ABO-Rh human blood typing. *Electrophoresis* **2011**; Vol. *32*, pp 2530-2540, doi:10.1002/elps.201100089.
24. Jen, C.P.; Chen, T.W. Selective trapping of live and dead mammalian cells using insulator-based dielectrophoresis within open-top microstructures. *Biomedical microdevices* **2009**; Vol. *11*, pp 597-607, doi:10.1007/s10544-008-9269-1.
25. Huang, C.-T.; Weng, C.-H.; Jen, C.-P. Three-dimensional cellular focusing utilizing a combination of insulator-based and metallic dielectrophoresis. *Biomicrofluidics* **2011**; Vol. *5*, pp 44101-4410111, doi:10.1063/1.3646757.
26. Rosales-Cruzaley, E.; Cota-Elizondo, P.A.; Sanchez, D.; Lapizco-Encinas, B.H. Sperm cells manipulation employing dielectrophoresis. *Bioprocess and biosystems engineering* **2013**; Vol. *36*, pp 1353-1362, doi:10.1007/s00449-012-0838-6.
27. Kikkeri, K.; Kerr, B.A.; Bertke, A.S.; Strobl, J.S.; Agah, M. Passivated-electrode insulator-based dielectrophoretic separation of heterogeneous cell mixtures. *Journal of Separation Science* **2020**; Vol. *n/a*, pp 1-10, doi:10.1002/jssc.201900553.
28. Liu, Y.; Jiang, A.; Kim, E.; Ro, C.; Adams, T.; Flanagan, L.A.; Taylor, T.J.; Hayes, M.A. Identification of neural stem and progenitor cell subpopulations using DC insulator-based dielectrophoresis. *Analyst* **2019**; Vol. *144*, pp 4066-4072, doi:10.1039/C9AN00456D.
29. Gallo-Villanueva, R.C.; Jesús-Pérez, N.M.; Martínez-López, J.I.; Pacheco, A.; Lapizco-Encinas, B.H. Assessment of microalgae viability employing insulator-based dielectrophoresis. *Microfluidics and Nanofluidics* **2011**; Vol. *10*, pp 1305-1315, doi:10.1007/s10404-010-0764-3.
30. Kim, D.; Luo, J.; Arriaga, E.A.; Ros, A. Deterministic Ratchet for Sub-micrometer (Bio)particle Separation. *Analytical Chemistry* **2018**; Vol. *90*, pp 4370-4379, doi:10.1021/acs.analchem.7b03774.
31. Mohammadi, M.; Madadi, H.; Casals-Terré, J.; Sellarès, J. Hydrodynamic and direct-current insulator-based dielectrophoresis (H-DC-iDEP) microfluidic blood plasma separation. *Analytical and bioanalytical chemistry* **2015**; Vol. *407*, pp 4733-4744, doi:10.1007/s00216-015-8678-2.
32. Davalos, R.V., Shafiee, Hadi, Sano, Michael Benjamin , Caldwell, John L. Devices and methods for contactless dielectrophoresis for cell or particle manipulation. U.S. Patent 20190137446 [P], 9 May 2019.
33. Swami; Nathan; Su; Yi-Hsuan; Warren; Alcantara, C.; Rohani; Ali; Farmehini; Vahid Identification and monitoring of cells by dielectrophoretic tracking of electrophysiology and phenotype. U.S. Patent 20170218424 A [P], 3 September 2017.

34. Davalos; Rafael V. , S.; Blake A. , C.; Robert W. , C.; B., E. Insulator-based DEP with impedance measurements for analyte detection. U.S. Patent 7678256 B2 [P], 26 January 2017.
35. Cummings, R.V.D.A.S.W.C.B. Insulator-Based DEP with Impedance Measurements for Analyte Detection. U.S. Patent 20080105565 A1 [P], 8 May 2008.
36. Cummings; Eric B. (Livermore, C., Fintschenko; Yolanda (Livermore, C., Simmons; Blake (San Francisco, C. Dielectrophoresis device and method having nonuniform arrays for manipulating particles. U.S. Patent 20190137446 [P]. U.S. Patent 7419574, 2 September 2008.
37. Masuda, T.; Maruyama, H.; Honda, A.; Arai, F. Virus enrichment for single virus infection by using 3D insulator based dielectrophoresis. *PLoS One* **2014**; Vol. *9*, pp e94083-e94083, doi:10.1371/journal.pone.0094083.
38. Coll De Peña, A.; Mohd Redzuan, N.H.; Abajorga, M.K.; Hill, N.; Thomas, J.A.; Lapizco-Encinas, B.H. Analysis of Bacteriophages with Insulator-Based Dielectrophoresis. *Micromachines (Basel)* **2019**; Vol. *10*, pp 450, doi:10.3390/mi10070450.
39. Lapizco-Encinas, B.H.; Davalos, R.V.; Simmons, B.A.; Cummings, E.B.; Fintschenko, Y. An insulator-based (electrodeless) dielectrophoretic concentrator for microbes in water. *Journal of Microbiological Methods* **2005**; Vol. *62*, pp 317-326, doi:10.1016/j.mimet.2005.04.027.
40. Ding, J.; Lawrence, R.M.; Jones, P.V.; Hogue, B.G.; Hayes, M.A. Concentration of Sindbis virus with optimized gradient insulator-based dielectrophoresis. *Analyst* **2016**; Vol. *141*, pp 1997-2008, doi:10.1039/C5AN02430G.
41. Davalos; Rafael V., S.; Hadi, S.; Michael B., C.; L., J. Devices and methods for contactless dielectrophoresis for cell or particle manipulation. U.S. patent 8968542 B2, 3 March 2015.
42. Morales; Alfredo M., W.; Josh A., Z.; Mark D. (Livermore, C., Renzi; Ronald F., T.; Maurer, H.M.; Scott M., M.; D., W. System for particle concentration and detection. U.S. patent 8398839 B1, 19 March 2013.
43. Simmons; Blake A., H.; Vincent R. , F.; Yolanda, C.; B., E. Method for concentration and separation of biological organisms by ultrafiltration and dielectrophoresis. U.S. patent 8257568 B1, 4 September 2012 .
44. Davalos; Rafael V., S.; Blake A., C.; Robert W., C.; B., E. Insulator-based DEP with impedance measurements for analyte detection. U.S. patent 7678256 B2, 26 January 2017.
45. Cummings; Eric B., F.; J., G. Dielectrophoresis device and method having insulating ridges for manipulating particles. U.S. patent 7347923 B2, 25 March 2008.
46. Cummings, E.B.; Singh, A.K. Dielectrophoretic systems without embedded electrodes. U.S. patent 20040026250 A1, 12 February 2004.
47. Bakajin., R.H.A.O.T.C.C.-F.C. Electrodeless dielectrophoresis for polarizable particles. France patent WO2001037958A2, 31 May 2001.

© 2020 by the authors. Submitted for possible open access publication under the terms and conditions of the Creative Commons Attribution (CC BY) license (http://creativecommons.org/licenses/by/4.0/).
